# Transcutaneous Electrical Acupoint Stimulation Reduces Postoperative Analgesic Requirement in Patients Undergoing Inguinal Hernia Repair: A Randomized, Placebo-Controlled Study

**DOI:** 10.3390/jcm10010146

**Published:** 2021-01-04

**Authors:** Mateusz Szmit, Siddarth Agrawal, Waldemar Goździk, Andrzej Kübler, Anil Agrawal, Piotr Pruchnicki, Marta Woźniak, Matylda Nowak, Bartłomiej Bartoszewicz, Jerzy Rudnicki

**Affiliations:** 1 Department and Clinic of General, Minimally Invasive and Endocrine Surgery, Wroclaw Medical University, 50-556 Wroclaw, Poland; mateusz.szmit@umed.wroc.pl (M.S.); jerzy.rudnicki@umed.wroc.pl (J.R.); 2 Department and Clinic of Internal Medicine, Occupational Diseases, Hypertension and Clinical Oncology, Wroclaw Medical University, 50-556 Wroclaw, Poland; 3 Department of Pathology, Wroclaw Medical University, 50-556 Wroclaw, Poland; marta.wozniak@umed.wroc.pl; 4 Department and Clinic of Anesthesiology and Intensive Therapy, Wroclaw Medical University, 50-556 Wroclaw, Poland; andrzej.kubler@umed.wroc.pl; 5 Second Department and Clinic of General and Oncological Surgery, Wroclaw Medical University, 50-556 Wroclaw, Poland; anil.agrawal@umed.wroc.pl; 6 Department of Acoustics and Multimedia, Faculty of Electronics, Wroclaw University of Science and Technology, 50-370 Wroclaw, Poland; piotr.pruchnicki@pwr.edu.pl; 7 Department of Design, The Eugeniusz Geppert Academy of Art and Design, 50-416 Wroclaw, Poland; m.nowak@asp.wroc.pl; 8 Department of Econometrics and Operations Research, Wroclaw University of Economics and Business, 53-345 Wroclaw, Poland; bbartoszewicz@gmail.com

**Keywords:** acupuncture, patient-controlled analgesia, TEAS, inguinal hernia

## Abstract

Given the rising rate of opioid-related adverse drug events during postsurgical pain management, a nonpharmacologic therapy that could decrease analgesic medication requirements would be of immense value. We designed a prospective, placebo-and-randomized controlled trial to assess the clinical effect of transcutaneous acupoint electrical stimulation (TEAS) on the postoperative patient-controlled analgesia (PCA) requirement for morphine, as well as side effects and recovery profile after inguinal hernia repair. Seventy-one subjects undergoing inguinal hernia repair with a standardized anesthetic technique were randomly assigned to one of three analgesic treatment regimens: PCA + TEAS (*n* = 24); PCA + sham-TEAS (no electrical stimulation) (*n* = 24), and PCA only (*n* = 23). The postoperative PCA requirement, pain scores, opioid-related side effects, and blood cortisol levels were recorded. TEAS treatment resulted in a twofold decrease in the analgesic requirement and decreased pain level reported by the patients. In addition, a significant reduction of cortisol level was reported in the TEAS group at 24 h postoperatively compared to the sham and control groups. We conclude that TEAS is a safe and effective option for reducing analgesic consumption and postoperative pain following inguinal hernia repair.

## 1. Introduction

Opioid analgesia remains the mainstay for postsurgical pain management, yet the consequences of its use incur risks for patients before and after discharge [1]. Opioid administration has been associated with increased hospital length of stay, treatment costs, readmissions, and mortality, particularly with higher doses of opioids [2,3]. Moreover, previously opioid-free patients are at an increased risk of developing prolonged opioid use when discharged with outpatient prescriptions for these analgesics [4]. Opioid-related adverse drug events (ORADEs) are common among patients undergoing hospital-based invasive procedures and are associated with significantly worse economic, as well as clinical outcomes, the most serious being respiratory depression [5]. Notably, adverse events can occur with any opioid, including morphine, oxycodone, hydrocodone, fentanyl, methadone, and sufentanil. Given the rising rate of opioid use disorder and opioid-related overdose in the clinical setting, evaluating a nonpharmacologic approach to managing postoperative pain, which could effectively reduce the opioid analgesic requirement, is of immense value.

Transcutaneous electrical acupoint stimulation (TEAS) is a non-invasive, safe, and comfortable treatment modality, which combines the effects of transcutaneous electrical nerve stimulation (TENS) with acupuncture point stimulation. TEAS excites the afferent nerves at an acupuncture point with low-voltage impulses. It is based, in part, on traditional Chinese analgesic methods and is known to suppress the transmission and perception of injurious stimuli [6]. The current evidence indicates that endogenous opioid peptides mediate the therapeutic effect, as electroacupuncture analgesia is reversed by the opioid receptor antagonist naloxone [7]. Moreover, an increase in endogenous opioids in plasma and cerebrospinal fluid have been reported in patients following electroacupuncture treatment [8]. The accumulating evidence suggests that TEAS exerts a clinically meaningful effect on postoperative pain control after various surgeries, including laparoscopic surgery, total hip arthroplasty, thoracoscopic surgery, breast surgery, hemorrhoidectomy, and total abdominal hysterectomy or myomectomy [9,10,11,12], which is proved to be superior to TENS (stimulation of nonacupoints) [13,14]. Given the relative advantages of TEAS over classical acupuncture and needle-based electrostimulation, which stem from its non-invasive character and the possibility of continuous, multiple stimulations, the modality emerges as a promising approach for the management of postoperative pain.

Most prior assessments have shown a beneficial effect of TENS or electroacupuncture (EA) in reducing pain intensity after hernia surgery [15,16,17]. However, to date, there are no randomized controlled trials to evaluate the efficacy of this non-pharmacologic modality on opioids consumption in patients undergoing inguinal hernia repair. The present prospective, randomized, sham-controlled, and single-blinded study was designed to assess the clinical effect of TEAS on the postoperative opioid analgesic requirement, as well as side effects and recovery profile in the early postoperative period after inguinal hernia repair.

## 2. Experimental Section

### 2.1. Study Design

This prospective, three-armed, single-blinded, placebo-controlled randomized controlled trial was conducted at Wroclaw University Hospital, Wroclaw, Poland, from September 2017 to November 2020. The study was conducted in accordance with the Helsinki Declaration to protect the participants and was approved by the ethics committee of Wroclaw Medical University (No. KB-599/2017). The study protocol was registered at the ISRCTN registry (ISRCTN76428396; https://doi.org/10.1186/ISRCTN76428396). All participants provided written informed consent before randomization and after being informed on the potential benefits, risks, alternatives, and responsibilities of the study.

### 2.2. Participants

#### 2.2.1. Inclusion Criteria

- Male and female patients aged 18–75 years
- Patients undergoing elective laparoscopic mesh inguinal hernia repair
- Body mass index 18–30 kg/m^2^
- ASA classification I–III
- Patients provide signed informed consent

#### 2.2.2. Exclusion Criteria

- Patients with bilateral or recurrent inguinal hernia
- Patients with a history of intolerance, hypersensitivity, or abuse of opioids
- Use of opioids in the past month
- Use of monoamine oxidase and selective serotonin reuptake inhibitors
- Patients wearing a cardiac pacemaker
- Patients with clinically significant cardiovascular, pulmonary, renal, hepatic, and neurological disease
- Patients with skin infections, surgical incision, or scar at the point of application of acupuncture
- Patients who participated in other clinical trials or received other acupuncture therapy in the previous four weeks

### 2.3. Randomization and Concealment

**Figure 1:**
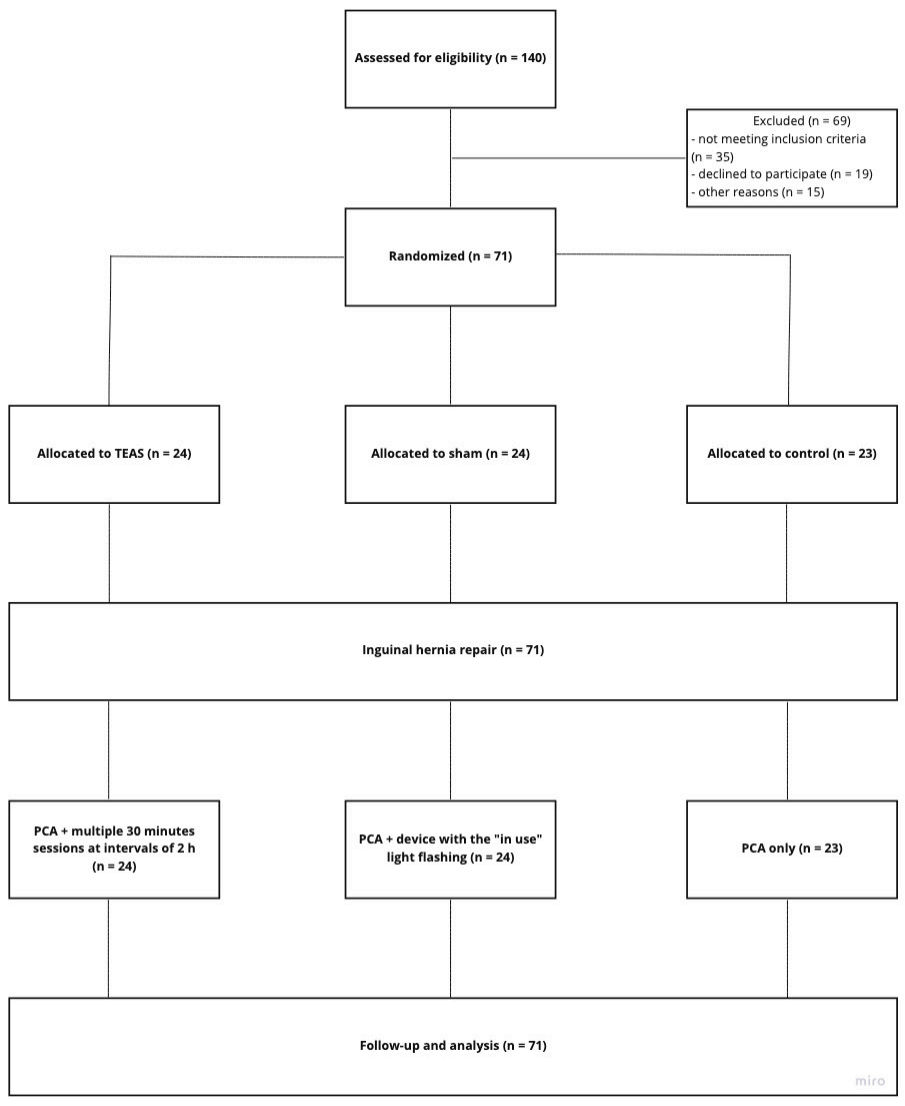
Schematic presentation of the study with special regard to the classification of enrolled participants. TEAS—transcutaneous acupoint electrical stimulation; PCA—patient-controlled analgesia.

Enrolled participants were randomly assigned to TEAS, sham, or control group (1:1:1). An independent, blinded statistician generated the block randomization scheme. The table was managed by an independent researcher who was not involved in the recruitment, treatment, or assessment. The participants were blinded to the type of treatment. Mock TEAS was provided with the same lamplight as real TEAS, so the participants were not able to predict the allocated group based on the appearance of the treatment (Figure 1).

### 2.4. Sample Size 

Power analysis was performed to determine the number of patients per group sufficient to detect a decrease of 30% or more in the PCA opioid analgesic requirements during the first 24 h after surgery, based on the results of the previous studies [13,18,19]. Each group included minimum 23 participants, a total of 71 participants. 

### 2.5. Interventions

#### 2.5.1. Anesthesia Protocol

A standard premedication of 7.5 mg oral midazolam was administered in every patient 30 min prior to entering the operating room. Thereafter, standard patient monitoring, including continuous electrocardiography, pulse oximetry, and indirect blood pressure (measured every 5 min), was applied and maintained until the end of anesthesia. Three-minute preoxygenation with 100% oxygen preceded the induction phase in every patient. Then, 1–3 mcg/kg fentanyl, followed by 2–3 mg/kg propofol and 0.6 mg/kg rocuronium, was applied through the peripheral vein to induce general anesthesia. Individually selected, an appropriate size endotracheal tube (ET) was placed via direct laryngoscopy. After confirmation of proper ET placement, mechanical ventilation was commenced, and anesthesia maintained by sevoflurane 2–4 vol.% mixed with 60% oxygen, titrated to achieve 0.9–1.2 MAC. Continuous monitoring of capnography was used to ensure proper respiratory function and gas exchange. During the surgery, additional boluses of fentanyl (1–2 mcg/kg) were administered in the presence of the patient’s reactions to pain stimuli (i.e., tachycardia/hypertension). Depending on the time of surgery, an additional bolus of 25% of the initial dose of rocuronium was applied. For basic pain control, 1 g of metamizole was given to every patient before completion of the surgery. After cessation of sevoflurane administration and achieving an adequate level of consciousness and responsiveness, the patient was extubated. After the surgery, every patient was monitored and carefully observed for at least 30 min in the recovery room.

The surgical technique was the Lichtenstein tension-free method. Hernia patients, according to the classification of Nyhus, were type II or IIIb.

#### 2.5.2. Postoperative Analgesia

On a patient’s arrival in the postanesthesia care unit, the PCA device was connected to the patient’s intravenous line and programmed to deliver 1 mL bolus doses of morphine (1 mg) “on-demand”, with a minimal lockout interval of 10 min and a maximum 4 h dose of 15 mg according to a standardized hospital protocol. PCA therapy was initiated in the postanesthesia care unit when the patient was sufficiently alert to understand and operate the PCA device. If the patient required pain medication prior to starting PCA therapy, an incremental dose of metamizole 1 g intravenously was administered by the postanesthesia care unit nursing staff. The postoperative PCA analgesic therapy was supplemented with TEAS/sham therapy, which started when the patient arrived in the postanesthesia care unit.

**Figure 2:**
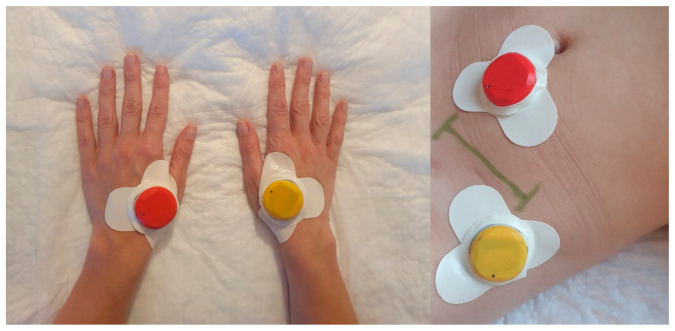
Distribution of four portable electro-simulators at enrolled participants.

Stimulation was performed using four portable coin-sized electro-stimulators (Zerniki Wroclawskie, StimulAid Inc., Poland). A point-detection function in the device confirmed the correct localization of the stimulator. The TEAS group received mixed frequency stimulation (alternating at 2 and 100 Hz every 3 s) in continuous mode for 30 min at intervals of 2 h. The device was automatically shut off at the end of each 30 min treatment interval. The intensity of the stimulation was adjusted to each individual to maintain a slight twitching of the regional muscle and achieve De-Qi sensations, such as soreness, distention, and heaviness. To minimize the arousal effect of electrical stimulation on the patient’s quality of rest and sleep, the intensity of stimulation was reduced by 50% during sleep hours. In both groups, electro-stimulators were applied bilaterally to LI4 (He Gu) and ipsilateral to hernia to two ashi points located within a diameter of 5 cm from the incision site (Figure 2). The points were selected based on the results of a literature review of previous studies examining the effect of electroacupuncture or TENS on postoperative pain management [19,20,21,22,23]. The sham group was provided with the same devices as TEAS with the “in use” light flashing in the usual manner; however, the participants were told that they might not be able to feel the electrical stimulation. The patients in TEAS and sham groups were told that they are receiving current stimulation. The device included a skin-electrode impedance measurement system, which facilitated the correct placement of the stimulator on the acupoint as well as the tracing of any displacement during the treatment. The quality of the connection was measured and recorded by the stimulator and transferred wirelessly to the application. The readings allowed the investigator to monitor the localization of the devices throughout the treatment period (Figure S1). In the case of displacement of the device, the investigator corrected the localization of the stimulator. The stimulator sends a pulse of the current of a predetermined width repeated at a specified frequency. The intensity of the current in the pulse is set individually for each patient in the range from 1 mA to 6 mA. The current level used in the study ranged from 1.2 mA to 2.5 mA (mean 1.85; SD 0.31). The PCA therapy and TEAS/sham were discontinued at 24 h.

### 2.6. Outcomes

#### 2.6.1. Primary Outcome

Total morphine dose received in the postoperative period (mg) using patient-controlled analgesia (PCA) device (B. Braun AG, Melsungen, Germany).

#### 2.6.2. Secondary Outcomes

The number of PCA demands (i.e., times the button was pressed) and delivered bolus doses after surgery. Score on the Visual Analogue Scale prior to the surgery and at 4, 8, 12, 16, and 20 h after surgery. The scale of 0 to 10, where 0 represents the complete absence of pain and 10 represents the worst pain intensity. Moreover, opioid-related side effects and the requirements for supplemental medications (e.g., antiemetics, antipruritics, and analgesics) were recorded during the postoperative observation period. 

### 2.7. Cortisol Levels Assessment

The patients’ whole blood was collected between 8 and 10 a.m. on the day before the surgery and 24-h later within the same timeframe. After collection and the samples were transferred to an appropriate collection tube. After being centrifuged at 10,000 rpm for 10 min, serum was transferred into a new cryotube to freeze the samples at −80 °C until the analysis. Cortisol level was detected by an enzyme immunoassay using the ELISA kit (Cortisol ELISA Kit; Thermo Fisher Scientific Inc., Waltham, MA, USA). The 5 µL of serum samples were resuspended in 5 µL Dissociation Reagent, and 490 µL of the Assay Diluent included in the ELISA kit to prepare final serum dilution 1:100. Then cortisol levels were analyzed according to the manufacturer’s instructions. The absorbance was measured at 490 nm on microtiter plate reader BioTek ELX800 (BioTek, Winooski, VT, USA). Cortisol levels were analyzed in a total of 21 patients (7 in each arm). The standards and samples were prepared in duplicate, and the results are presented as means. Intra-assay variances between data points within an assay were between 1 and 5%. The levels of cortisol are shown as ng/mL. 

### 2.8. Adverse Events

All adverse events were closely monitored through reports by participants or direct observation by personnel and by asking the patients about adverse events during the observation period. All adverse reactions were recorded, and additional treatment was offered if required. 

### 2.9. Statistical Analysis

Quantitative variables were reported using the mean and SD (M ± SD) for normally distributed data or median (Me), the lower quartile (Q1), and the upper quartile (Q3) and the range (min–max) for skewed data. Shapiro–Wilk’s test was used to verify the normality of distributions. In the one-way analysis, the Student’s *t*-test (*t*-test for Independents Samples by Groups) was used to compare the mean values in two groups, and in the case of three groups-one-way analysis of variance (ANOVA). HSD Tukey’s test (Tukey honest significant difference) and Kruskal–Wallis ANOVA were used for multiple comparisons (post hoc tests). In addition, a multivariate analysis, ANOVA for factorial designs (Factorial ANOVA), was performed where the main effects and their interactions (relationships between independent variables) were analyzed. The significance level was set at 5%. All data were analyzed using Statistica v.13.3 (TIBCO Software Inc., Palo Alto, CA, USA). 

### 2.10. Data Availability

The datasets analyzed for the present study were collected directly from the patients and will not be publicly shared due to conditions of ethical approval. The datasets are available from the author upon reasonable request.

## 3. Results

### 3.1. Participants and Baseline Characteristic

The recruitment and follow-up of the trial participants are presented in Figure 1. Of 140 patients screened, 71 patients were randomly assigned to the TEAS, Sham, or control group. The study included 60 men (84.5%) and 11 women (15.5%) aged 23 to 89 years (mean 57.6; standard deviation SD = 14.6 years). The baseline characteristic of the randomized groups is presented in Table 1. All three patient groups were homogeneous in terms of gender and age (*p* > 0.05).

### 3.2. Outcome Comparison between the Groups

The total morphine dose (mg) received in the postoperative period using PCA device in the first 24 h was significantly decreased in the TEAS group (8; 5–9, median and interquartile range, respectively) compared with the sham (15; 13–18) and control groups (15; 11–18) (*p* < 0.001; Table 2). 

**Figure 3:**
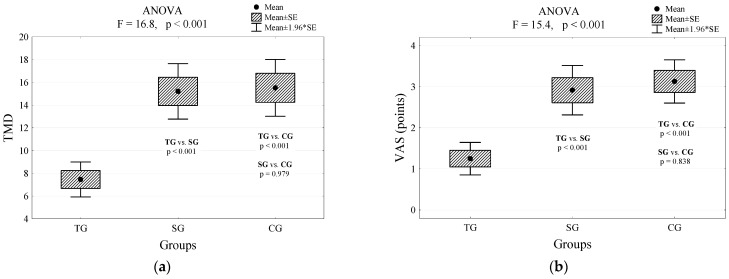

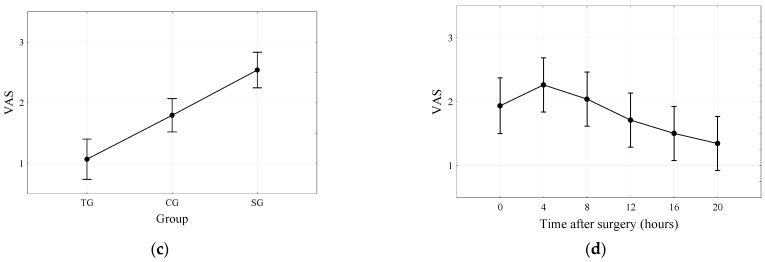
Differences in values of the level of perceived pain between enrolled participants: (**a**) Total dose of morphine consumed by patients with a different type of intervention and the result of the analysis of variance and multiple comparisons (Tukey’s post hoc tests); (**b**) Pain sensation assessed on the VAS scale at discharge by patients differing in the type of intervention and the result of the analysis of variance and multiple comparisons (Tukey’s post hoc tests); (**c**) Expected boundary values of the perceived pain (mean and their 95% confidence intervals) and the assessment of the impact of treatment; *p* < 0.00001; (**d**) Expected boundary values of the perceived pain (mean and their 95% confidence intervals) and time after the surgery; *p* = 0.028. TG—true group; CG—control group; SG—sham group; VAS—visual analog scale; TMD—total morphine dose in milligrams.

However, we found no statistical significance between the sham and control group (*p* = 0.979; Figure 3a). Interestingly, the pain VAS showed significantly lower scores in the TEAS group compared with the sham and control groups (*p* < 0.001; Table 2). No statistical significance was found between the sham and control group (*p* = 0.838; Figure 3b). The results of the two-way analysis of variance confirmed that the level of perceived pain in the study group was significantly lower than in the other groups of patients (Figure 3c), and all patients experienced the greatest pain at 4 h after the surgery (Figure 3d). 

**Figure 4:**
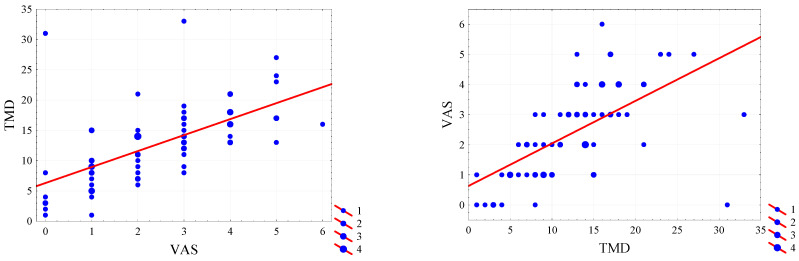
Correlation diagram between perceived pain and dose of morphine, Pearson’s correlation coefficient, and the regression line equation. VAS—visual analog scale (pain intensity quantified in points); TMD—total morphine (dose in milligrams).

A strong positive correlation was observed between the level of perceived pain and the dose of morphine administered (r = 0.723; Figure 4). With an increase in perceived pain (VAS) by 1 point, the dose of morphine (TMD) was increased by an average of 2.64 units (Figure 4).

Although there were no statistically significant differences in the presence of nausea among the three treatment groups (Table 2), there were no cases with nausea in the TEAS group. We found no statistically significant differences between the level of reported pain on the VAS scale between men and women (*p* = 0.190; Table 3).

There were statistically significant differences between TEAS and sham and control groups in cortisol measurements. At baseline, we found no statistically significant differences in cortisol levels between the groups; however, at 24 h postoperatively, cortisol in the TEAS group was significantly decreased compared with sham and control groups (*p* = 0.001; Table 4). 

**Figure 5:**
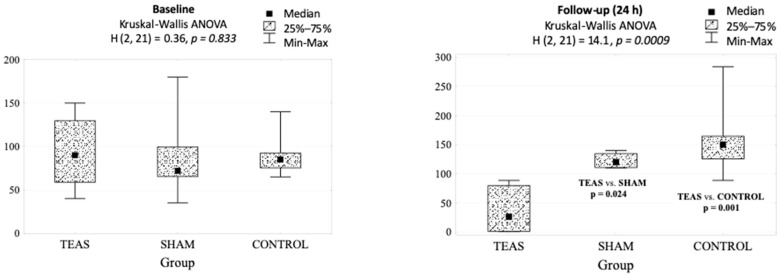
Cortisol levels (ng/mL) at baseline and follow-up in groups differing in the type of intervention and the result of the analysis of variance and multiple comparisons (Kruskal–Wallis ANOVA).

In addition, we have found that in the control group, the cortisol level was significantly higher at 24 h than preoperatively (*p* = 0.018). Although we have also observed a reduction in the cortisol level at follow-up compared to baseline in the study group, the difference did not reach statistical significance (*p* = 0.063). In TEAS-treated patients, the cortisol level at 24 h postoperatively was significantly lower compared to sham (*p* = 0.024) and control group (*p* = 0.001) (Figure 5).

On the follow-up interview, nearly 90% of the study patients reported that their postoperative pain was “satisfactorily” treated, and there were no significant differences among the three groups. No patient complained that the electrical stimulation produced by the TEAS device was uncomfortable. In one patient in the study group and two patients in the sham group, skin irritation (redness and itching) was observed; however, the severity of these symptoms was mild, and no subjects reported discomfort nor required specific treatment. Sedation, pruritus, vomiting, and other side-effects were not reported.

## 4. Discussion

With more than 20 million patients yearly, inguinal hernia repair is among the most commonly performed surgeries worldwide [24]. The procedure is characterized by a high intensity of postoperative pain and patient discomfort associated with surgical manipulation or placement of the preperitoneal mesh [25]. A mounting body of evidence suggests that acupuncture and acupuncture-related techniques can be performed before, during, or after surgery to alleviate postoperative pain [26]. TEAS or acupuncture-like TENS become more acceptable than acupuncture, given its non-invasive character and similar analgesic effect [27,28]. 

The results of this study clearly document the opioid-sparing effect of TEAS. We have found that the use of TEAS resulted in over 50% decrease in narcotic analgesic usage. Moreover, our results indicate that the level of pain was significantly lower in the study group compared with the sham and control groups. 

In the present study, a combination of the LI4 acupuncture point and peri-incisional ashi points were found to augment PCA analgesia in the postoperative period (Figure 2). Our results echo those of an earlier study that utilized both distal and local points. Several trials have used only distal points, including LI4 “Hegu” and ST36 “Zusanli” [20,21,22], or only local points around the incision region [13]; however, a combination of points prove to exert a strong analgesic effect [19,23]. Numerous clinicians assume that direct electrical stimulation around the incision region may lead to adverse events, but to date, there has not been any evidence of harmful effects such as increased pain or infection. 

Not only points selection but also the frequency of stimulation and treatment timing play a critical role in determining the outcome of the intervention. In humans, low-frequency (2 Hz) electrostimulation applied at the acupoints exerts antinociceptive effects mainly by enhancing the release of met-enkephalin (M-ENK) and β-endorphin (β-EP) [29,30]. The impact on pain tends to last longer than the electrostimulation itself. In contrast, high-frequency (100 Hz) stimulation produces a potent analgesic effect mediated by the release of dynorphin-A (DYN-A) in the central nervous system [31]. The effect, however, disappears shortly after discontinuing the stimulation. Experimental studies have shown that stimulation at 2 Hz and 100 Hz alternatively elicited the full release of M-ENK, β-EP, and DYN-A in the CNS, which produced a synergistic effect stronger than that at 2 Hz or 100 Hz alone [32,33]. Clinical practice proves that alternating stimulation with low and high frequencies can also induce a more potent analgesic effect [19]. Therefore, we elected to use alternating 2 and 100 Hz frequency stimulation in the present clinical investigation. 

Pain initially stimulates the hypothalamic-pituitary-adrenal system to produce cortisol and travel to tissue targets, including wounds and injured nerves. Therefore, in the stimulation phase of acute pain, cortisol levels are elevated [34]. Our study found that TEAS treatment resulted in a decrease of cortisol level at 24 h postoperatively compared to sham and control groups. It is assumed that TEAS analgesia is fundamentally a manifestation of integrative processes at various levels of the central nervous system between the afferent impulses from the pain loci and impulses from acupoints [35]. The statistically significant changes in the cortisol values strongly support the view that acupoint stimulation exerts its action through the neuroendocrine system. These findings corroborate those of the Dalamagka et al., which also investigated the effect of acupoint stimulation on stress hormones [16].

Duration of electrical stimulation can influence the effectiveness of TEAS. Continuous stimulation leads to the development of accommodation (or habituation) to the electrical current [36]. In contrast, if the interval between each session is excessive, the analgesic effect may be decreased because of the insufficient release of therapeutic mediators. Therefore, in our study, we elected the intermittent use of 30 min stimulation sessions for relatively short intervals (2 h) to minimize tolerance and achieve longer-lasting analgesia. Moreover, to decrease electrical stimulation’s arousal effect on the patient’s quality of rest and sleep, the intensity of stimulation was reduced by 50% during sleep hours. To the best of our knowledge, this is the first study that examined an intensified stimulation protocol with TEAS in reducing analgesic consumption after surgery. 

Interestingly, no patients in the study group reported that stimulation adversely influenced their quality of hospitalization. Notably, an earlier study has shown that over a third of all patients complained that cutaneous electrodes and wires had an adverse impact on their quality of sleep [19]. We have utilized four portable coin-sized electro-stimulators that ensured comfort to the patients during the 24 h-long treatment process. 

Since it has been implied that acupoint stimulation exerts a placebo effect with respect to its analgesic qualities [33], it was critical to blind the patient with respect to the treatment modality. We have performed a single-blind, sham-controlled study to eliminate the impact of patient bias on the results. Although a double-blind study design would have been preferable, due to the limits of the existing technology, we are unable to blind medical personnel. We believe that it is of great potential to utilize identical pre-programmed devices for sham and real group to minimize the investigator bias. In our study, the results indicate that the opioid analgesic dose requirements in the sham and control groups were not significantly different.

There are some limitations to this study. First, we only collected data on pain control in the short-term postoperative period. The effect of TEAS on long-term pain management after surgery needs further investigation. Second, although we tried to minimize the risk of patient bias by setting up a sham-group and providing the participants with the same devices as the true group, the limitation included lack of proper electrical stimulation. Third, it is necessary to study the effect of TEAS on postoperative analgesic consumption and pain control in large-scale multi-center studies and other types of surgery in the future.

Another factor analyzed in the study included the incidence of nausea after the surgery. Although there were no subjects reporting nausea in the study group, no statistically significant difference was found between the three arms of the study. The results are presumably secondary to the relatively low incidence of nausea in the trial. 

In conclusion, the postoperative use of TEAS resulted in a twofold decrease in the analgesic requirement. In addition, when TEAS was used as an adjunct to PCA, it produced a significant decrease in the level of pain reported by the patients. The evidence suggests that acupoint stimulation exerts its action through the neuroendocrine system. Adding the fact that it is easy to employ TEAS in a postoperative setting and that multiple stimulation sessions are possible, we conclude that TEAS is a safe and effective option for reducing analgesic consumption and postoperative pain following inguinal hernia repair. Further large-scale prospective randomized controlled trials are warranted to establish the TEAS-based treatment regimens for enhanced recovery after surgery.

## 5. Patents

MS, SA, PP, AA, WG, BB, MN, and JR are inventors on submitted patent applications (serial numbers P.431427, P.433134, and P.431878).

## Figures and Tables

**Table 1:** Baseline characteristics of patients in randomized groups.

Characteristic	Group						*p*-Value
	TEAS		Sham		Control		
	*n* = 24		*n* = 24		*n* = 23		
Gender	*n*	%	*n*	%	*n*	%	0.881
Females	3	12.5%	4	16.7%	4	17.4%	
Males	21	87.5%	20	83.3%	19	82.6%	
Age (years)							0.408
M (SD)	54.6 (15.8)		57.8 (15.6)		60.6 (12.1)		
Me (IQR)	55 (43; 68)		63 (43; 70)		63 (56; 69)		
Min–Max	27–74		23–74		27–75		

TEAS—transcutaneous acupoint electrical stimulation; SD—standard deviation; IQR—interquartile range.

**Table 2:** VAS pain sensation and total morphine dose (TMD).

Characteristic	Group						*p*-Value
	TEAS		Sham		Control		
VAS (points)							<0.001
M (SD)	1.3 (1.0)		2.9 (1.5)		3.1 (1.3)		
Me (IQR)	1 (0.5; 2)		3 (2; 4)		3 (2; 4)		
Min–Max	0–3		0–5		1–6		
TMD (mg):							<0.001
M (SD)	7.5 (3.8)		15.2 (6,24)		15.5 (6.1)		
Me (IQR)	8 (5; 9)		15 (13; 18)		15 (11; 18)		
Min–Max	1–18		1–31		4–33		
Nausea	*n*	%	*n*	%	*n*	%	0.116
No	24	100,0%	20	83.3%	21	91.3%	
Yes	0	0,0%	4	16.7%	2	8.7%	

TEAS—transcutaneous acupoint electrical stimulation; VAS—visual analog scale; TMD—total morphine dose; SD—standard deviation; IQR—interquartile range.

**Table 3:** Pain sensation on the VAS scale in groups of patients of different sex.

Characteristic	Gender		*p*-Value
	Females	Males	
	*n* = 11	*n* = 60	
VAS (points)			0.190
M (SD)	3.0 (1.7)	2.3 (1.5)	
Me (IQR)	3 (2; 5)	2 (1; 3)	
Min–Max	0–5	0–6	

VAS—visual analog scale; SD—standard deviation; IQR—interquartile range.

**Table 4:** Cortisol level (ng/mL) at baseline and at 24 h after the intervention.

	Group			*p*-Value
	TEAS	Sham	Control	
Baseline	90 (40–150)	72 (35–180)	85 (65–140)	0.833
Follow-up (24 h)	26 (1–89)	120 (110–140)	150 (89–284)	0.001
*p*-value	0.063	0.128	0.018	

TEAS—transcutaneous acupoint electrical stimulation.

## Data Availability

The data presented in this study are available on request from the corresponding author. The data are not publicly available due to privacy/ethical restrictions.

## Supplementary Materials

The following are available online at https://www.mdpi.com/2077-0383/10/1/146/s1, Figure S1: Monitoring of the location of acupuncture sites visible on the stimulator’s screen.

## References

[1] Brummett C.M., Waljee J.F., Goesling J., Moser S., Lin P., Englesbe M.J., Bohnert A.S.B., Kheterpal S., Nallamothu B.K. (2017). New persistent opioid use after minor and major surgical procedures in us adults. JAMA Surg..

[2] Oderda G.M., Said Q., Evans R.S., Stoddard G.J., Lloyd J., Jackson K., Rublee D., Samore M.H. (2007). Opioid-related adverse drug events in surgical hospitalizations: Impact on costs and length of stay. Ann. Pharmacother..

[3] Urman R.D., Seger D.L., Fiskio J.M., Neville B.A., Harry E.M., Weiner S.G., Lovelace B., Fain R., Cirillo J., Schnipper J.L. (2019). The Burden of Opioid-Related Adverse Drug Events on Hospitalized Previously Opioid-Free Surgical Patients. J. Patient Saf..

[4] Hah J.M., Bateman B.T., Ratliff J., Curtin C., Sun E. (2017). Chronic Opioid Use after Surgery: Implications for Perioperative Management in the Face of the Opioid Epidemic. Anesth. Analg..

[5] Shafi S., Collinsworth A.W., Copeland L.A., Ogola G.O., Qiu T., Kouznetsova M., Liao I.C., Mears N., Pham A.T., Wan G.J. (2018). Association of opioid-related adverse drug events with clinical and cost outcomes among surgical patients in a large integrated health care delivery system. JAMA Surg..

[6] Lai H.C., Lin Y.W., Hsieh C.L. (2019). Acupuncture-Analgesia-Mediated Alleviation of Central Sensitization. Evidence Complement. Altern. Med..

[7] Pomeranz B., Chiu D. (1976). Naloxone blockade of acupuncture analgesia: Endorphin implicated. Life Sci..

[8] Sjölund B., Terenius L., Eriksson M. (1977). Increased Cerebrospinal Fluid Levels of Endorphins after Electro-Acupuncture. Acta Physiol. Scand..

[9] Sun K., Xing T., Zhang F., Liu Y., Li W., Zhou Z., Fang L., Yu L., Yan M. (2017). Perioperative transcutaneous electrical acupoint stimulation for postoperative pain relief following laparoscopic surgery. Clin. J. Pain.

[10] Lan F., Ma Y.H., Xue J.X., Wang T.L., Ma D.Q. (2012). Transcutaneous electrical nerve stimulation on acupoints reduces fentanyl requirement for postoperative pain relief after total hip arthroplasty in elderly patients. Minerva Anestesiol..

[11] Song B., Chang Y., Li Y., Zhu J. (2020). Effects of transcutaneous electrical acupoint stimulation on the postoperative sleep quality and pain of patients after video-assisted thoracoscopic surgery: A prospective, randomized controlled trial. Nat. Sci. Sleep.

[12] Zhang Q., Gao Z., Wang H., Ma L., Guo F., Zhong H., Xiong L., Wang Q. (2014). The effect of pre-treatment with transcutaneous electrical acupoint stimulation on the quality of recovery after ambulatory breast surgery: A prospective, randomised controlled trial. Anaesthesia.

[13] Chen L., Tang J., White P.F., Sloninsky A., Wender R.H., Naruse R., Kariger R. (1998). The effect of location of transcutaneous electrical nerve stimulation on postoperative opioid analgesic requirement: Acupoint versus nonacupoint stimulation. Anesth. Analg..

[14] Chiu J.H., Chen W.S., Chen C.H., Jiang J.K., Tang G.J., Lui W.Y., Lin J.K. (1999). Effect of transcutaneous electrical nerve stimulation for pain relief on patients undergoing hemorrhoidectomy: Prospective, randomized, controlled trial. Dis. Colon Rectum.

[15] Dias M., Carneiro N.M., Vanni Guerra L.A., Velarde G.C., Teixeira De Souza P.A., Damásio Da Silva L.L., De Abreu E., Souza R.R., Nolasco R., Olej B. (2010). Effects of electroacupuncture on local anaesthesia for inguinal hernia repair: A randomised placebo-controlled trial. Acupunct. Med..

[16] Dalamagka M., Mavrommatis C., Grosomanidis V., Karakoulas K., Vasilakos D. (2015). Postoperative analgesia after low-frequency electroacupuncture as adjunctive treatment in inguinal hernia surgery with abdominal wall mesh reconstruction. Acupunct. Med..

[17] DeSantana J.M., Santana-Filho V.J., Guerra D.R., Sluka K.A., Gurgel R.Q., da Silva W.M. (2008). Hypoalgesic Effect of the Transcutaneous Electrical Nerve Stimulation Following Inguinal Herniorrhaphy: A Randomized, Controlled Trial. J. Pain.

[18] Hamza M.A., White P.F., Ahmed H.E., Ghoname E.S.A. (1999). Effect of the frequency of transcutaneous electrical nerve stimulation on the postoperative opioid analgesic requirement and recovery profile. Anesthesiology.

[19] Wang B., Tang J., White P.F., Naruse R., Sloninsky A., Kariger R., Gold J., Wender R.H. (1997). Effect of the intensity of transcutaneous acupoint electrical stimulation on the postoperative analgesic requirement. Anesth. Analg..

[20] Lin J.G., Lo M.W., Wen Y.R., Hsieh C.L., Tsai S.K., Sun W.Z. (2002). The effect of high and low frequency electroacupuncture in pain after lower abdominal surgery. Pain.

[21] Ng S.S.M., Leung W.W., Mak T.W.C., Hon S.S.F., Li J.C.M., Wong C.Y.N., Tsoi K.K.F., Lee J.F.Y. (2013). Electroacupuncture reduces duration of postoperative ileus after laparoscopic surgery for colorectal cancer. Gastroenterology.

[22] Wong R.H.L., Lee T.W., Sihoe A.D.L., Wan I.Y.P., Ng C.S.H., Chan S.K.C., Wong W.W.L., Liang Y.M., Yim A.P.C. (2006). Analgesic Effect of Electroacupuncture in Postthoracotomy Pain: A Prospective Randomized Trial. Ann. Thorac. Surg..

[23] Sim C.K., Xu P.C., Pua H.L., Zhang G., Lee T.L. (2002). Effects of electroacupuncture on intraoperative and postoperative analgesic requirement. Acupunct. Med..

[24] Simons M.P., Smietanski M., Bonjer H.J., Bittner R., Miserez M., Aufenacker T.J., Fitzgibbons R.J., Chowbey P.K., Tran H.M., Sani R. (2018). International guidelines for groin hernia management. Hernia.

[25] Callesen T. (2003). Inguinal hernia repair: Anaesthesia, pain and convalescence. Dan. Med. Bull..

[26] Wu M.S., Chen K.H., Chen I.F., Huang S.K., Tzeng P.C., Yeh M.L., Lee F.P., Lin J.G., Chen C. (2016). The efficacy of acupuncture in post-operative pain management: A systematic review and meta-analysis. PLoS ONE.

[27] Chesterton L.S., Martyn Lewis A., Sim J., Mallen C.D., Mason E.E., Hay E.M., Van Der Windt D.A. (2013). Transcutaneous electrical nerve stimulation as adjunct to primary care management for tennis elbow: Pragmatic randomised controlled trial (TATE trial). BMJ.

[28] Gavronsky S., Koeniger-Donohue R., Steller J., Hawkins J.W. (2012). Postoperative Pain: Acupuncture versus Percutaneous Electrical Nerve Stimulation. Pain Manag. Nurs..

[29] Han J.S., Chen X.H., Sun S.L., Xu X.J., Yuan Y., Yan S.C., Hao J.X., Terenius L. (1991). Effect of low- and high-frequency TENS on Met-enkephalin-Arg-Phe and dynorphin A immunoreactivity in human lumbar CSF. Pain.

[30] Han J.S. (2003). Acupuncture: Neuropeptide release produced by electrical stimulation of different frequencies. Trends Neurosci..

[31] Wang Y., Zhang Y., Wang W., Cao Y., Han J.S. (2005). Effects of synchronous or asynchronous electroacupuncture stimulation with low versus high frequency on spinal opioid release and tail flick nociception. Exp. Neurol..

[32] Xiao-Hong C., Su-Fong G., Chung-Gwo C., Ji-Sheng H. (1994). Optimal conditions for eliciting maximal electroacupuncture analgesia with dense-and-disperse mode stimulation. Am. J. Acupunct..

[33] Sun Y., Gan T.J., Dubose J.W., Habib A.S. (2008). Acupuncture and related techniques for postoperative pain: A systematic review of randomized controlled trials. Br. J. Anaesth..

[34] Tennant F. (2013). The Physiologic Effects of Pain on the Endocrine System. Pain Ther..

[35] Zhao Z.Q. (2008). Neural mechanism underlying acupuncture analgesia. Prog. Neurobiol..

[36] Chernyak G.V., Sessler D.I. (2005). Perioperative acupuncture and related techniques. Anesthesiology.

