# Supplementary figures and images for: Transcutaneous Electrical Acupoint Stimulation Reduces Postoperative Analgesic Requirement in Patients Undergoing Inguinal Hernia Repair: A Randomized, Placebo-Controlled Study
Source: J Clin Med. 2021 Jan 4;10(1):146. doi: 10.3390/jcm10010146 (PMC7794768; doi:10.3390/jcm10010146)

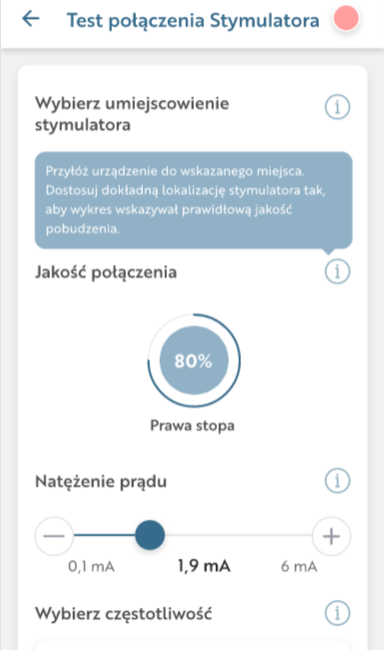

Supplement: Supplementary file 1 [file jcm-10-00146-s001.zip › jcm-1051109-supplementary.png]
